# Co-occurrence of aortic dissection and polycystic kidney disease: a case report

**DOI:** 10.1186/s13256-026-06658-9

**Published:** 2026-10-21

**Authors:** Ganesh Raj, Joseph Westaby, Mary N. Sheppard

**Affiliations:** 1 CRY Cardiovascular Pathology Unit, Cardiovascular Clinical Academic Group, Molecular and Clinical Sciences Research Institute, St. George’s, University of London, SW17 0RE London, United Kingdom; 2 Ministry of Health, Putrajaya, Malaysia

**Keywords:** Aortic dissection, Polycystic kidney disease, Sudden death, Teenager, Forensic pathology

## Abstract

**Background:**

Aortic dissection is a rare and often fatal condition which arises when there is aortic wall weakening. Multiple risk factors could lead to this condition, including hypertension, atherosclerosis, connective tissue diseases such as Marfan’s, Ehlers–Danlos and Loeys Dietz syndromes, as well as smoking, cocaine usage, bicuspid aortic valve, pregnancy and trauma. Polycystic kidney disease (PKD) is a rare condition characterized by the development of multiple renal cysts, which is caused by genetic abnormalities leading to autosomal dominant or autosomal recessive inheritance. However, the presentation of both pathologies simultaneously is rare and has been infrequently reported.

**Case presentation:**

We report a death attributed to the co-occurrence of both conditions, involving a 19-year-old Caucasian male with no known past medical history, who collapsed during sexual intercourse. Post-mortem examination revealed hemopericardium with an aortic dissection, which was associated with PKD. The heart and the great vessels were sent to the author's place of practice for a detailed cardiovascular pathology examination.

**Conclusion:**

This case is a reminder to all forensic and clinical pathologists that an extremely uncommon manifestation can occur; therefore, a meticulous approach should always be adopted before corroborating a cause of death, especially in young individuals. The relationship between both conditions in this case remains uncertain; however, PKD may represent an associated finding and possibly a predisposing factor rather than a direct causal mechanism. The co-occurrence of both pathologies raises the likelihood of an underlying predisposition to vascular pathology and may have implications for the screening and clinical evaluation of surviving blood relatives.

## Background

Aortic dissection is characterized by the separation of the tunica intima and media with tracking of blood longitudinally. Most often, an intimal tear is associated with a dissection at the weakened area on the aortic wall. The dissection can be transverse, vertical or T-shaped, which usually occurs 2 cm above the aortic valve on the ascending aorta [1]. According to the investigation by the Healthcare Safety Investigation Branch, there are about 2500 cases per year in the United Kingdom, and 20–30% of patients die before reaching the hospital [2].

Polycystic kidney disease (PKD) is an inherited condition. It leads to the formation of multiple fluid-filled non-cancerous cysts of varying sizes. There are two forms of PKD: autosomal dominant (ADPKD), usually seen in people aged between 30 and 60 years, and autosomal recessive (ARPKD), seen after birth or during childhood. Roughly 70,000 people have PKD in the United Kingdom, including children and 1 in 20,000 babies are born with ARPKD [3].

We report a case of sudden death in a teenager with no reported antecedent symptoms, who collapsed during sexual intercourse. The post-mortem examination revealed the co-occurrence of aortic dissection and PKD. This case is noteworthy because of the unusually young age of the decedent compared with most reported cases, the absence of known medical history, and the incidental discovery of both pathologies during post-mortem examination, contributing to the cause of death.

This case highlights the importance of considering underlying hereditary disorders and occult cardiovascular pathology in unexpected sudden deaths among apparently healthy young individuals, particularly when death occurs suddenly during exertion.

## Case presentation

The deceased was a 19-year-old Caucasian male, measuring 175 cm in height, weighing 90 kg, and having a body mass index (BMI) of 29 kg/m^2^ with no past medical history. Family and psychosocial history were unavailable, which has limited further assessment of possible hereditary risk factors, preceding symptoms, and relevant psychosocial circumstances.

He was having sexual intercourse before his collapse. The death was confirmed to be non-suspicious and non-traumatic, and a post-mortem examination was conducted. Toxicology screening for alcohol and drugs was negative. External examination revealed no obvious injuries or abnormalities.

**Fig. 1:**
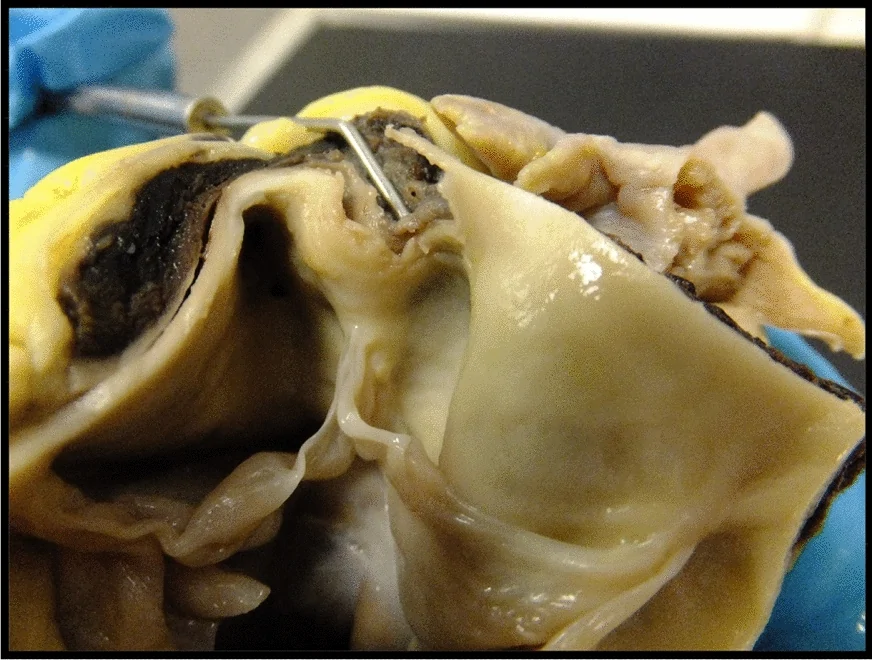
Dissected area above the non-coronary sinus

**Fig. 2:**
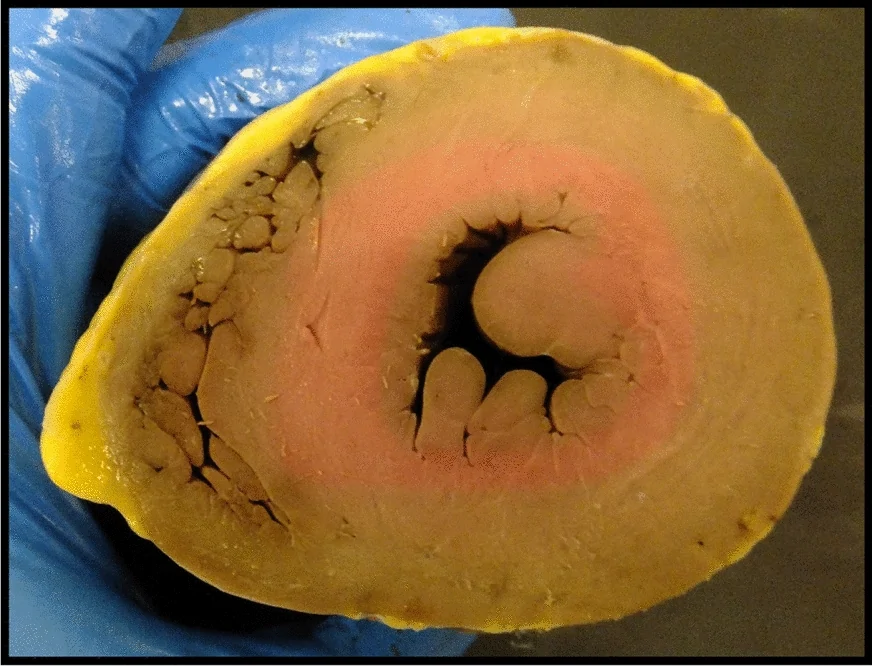
Symmetrical left ventricular wall thickening with no infarct or fibrosis

The internal examination revealed a blue dome appearance of the pericardial sac containing 600 ml of blood and blood clots. The ascending aorta was dilated with a circumference of 85 mm. An acute dissection measuring 15 mm in length above the non-coronary sinus (Fig. 1) was seen. The dissection plane extended 30 mm, involving half the circumference of the aorta, but no involvement of the coronary arteries was seen. The aortic valve was trileaflet and unremarkable. The heart weighed 456 gm, and the left ventricle revealed symmetrical wall thickening measuring 20 mm (Fig. 2). Otherwise, the coronary arteries, valves and myocardium were unremarkable. The kidneys revealed evidence of PKD, whereas the other organs were unremarkable on gross examination.

**Fig. 3:**
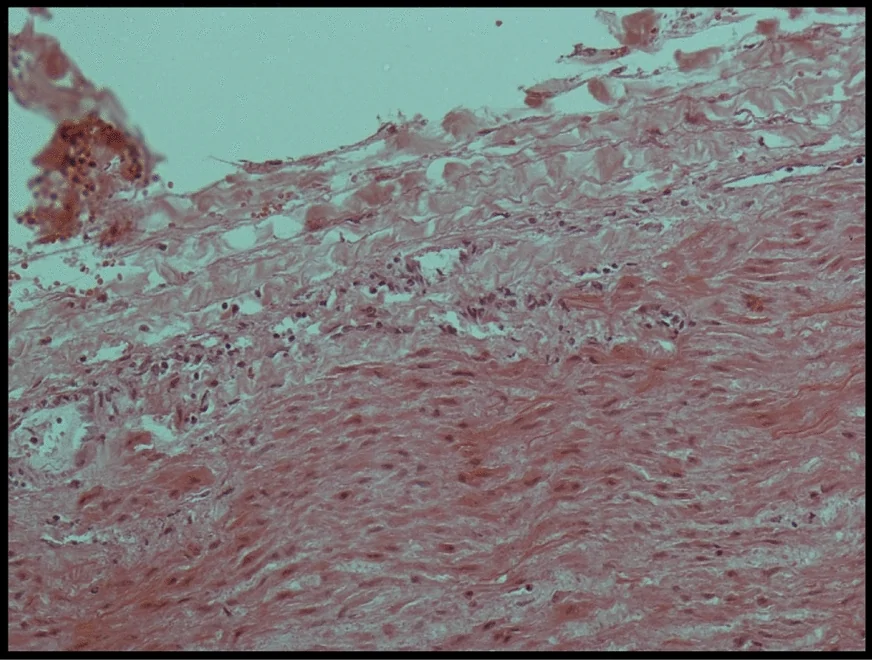
Area of dissection with thrombus and acute inflammatory cell infiltrations

**Fig. 4:**
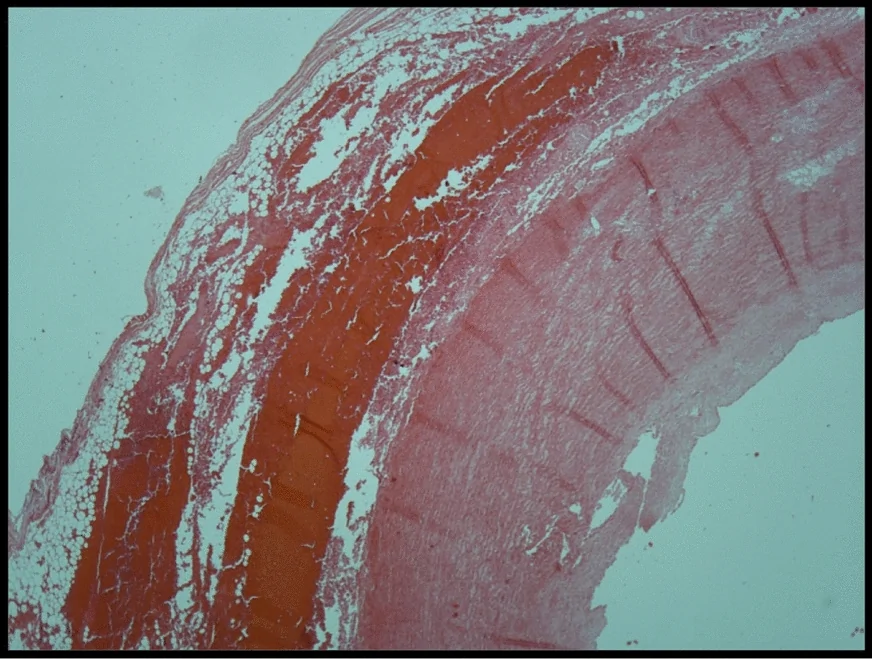
Area of dissection with thickened tunica intima and severe medial degenerative changes in hematoxylin and eosin (H&E) staining

**Fig. 5:**
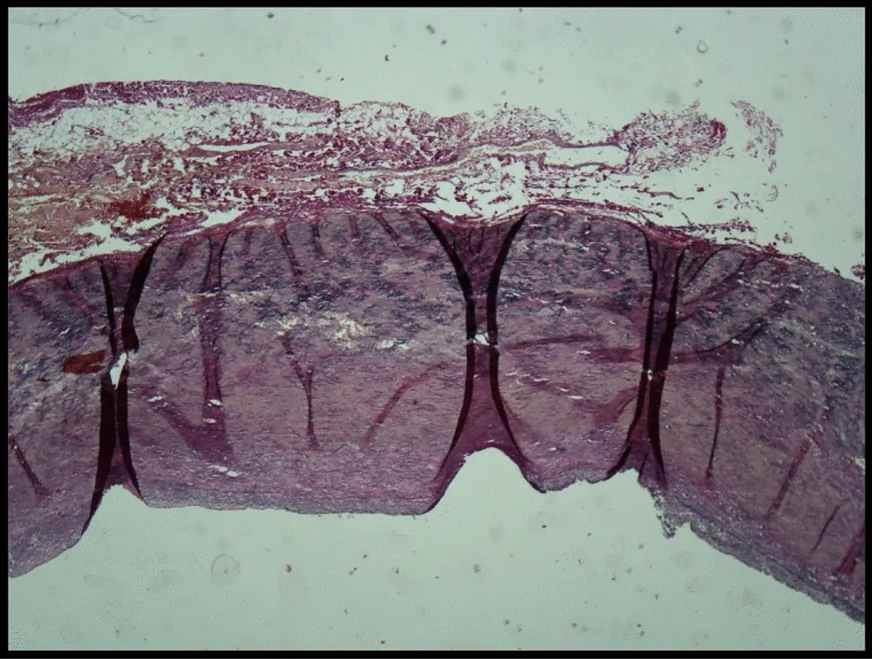
Medial degenerative changes in Elastica Van Gieson (EVG) staining

Microscopic examination revealed left ventricular myocyte hypertrophy, but no evidence of myocardial disarray to indicate hypertrophic cardiomyopathy. The aorta showed an acute dissection plane with adherent thrombus and acute inflammatory cell infiltration (Fig. 3). The intima shows minimal thickening (Fig. 3), and the media shows extracellular matrix accumulation and extensive elastic fiber loss in keeping with severe medial degenerative change (Figs. 4, 5), whereas the adventitia shows hemorrhage. There was no evidence of aortitis or other significant histopathological changes in the other organs.

Microscopic examination of the kidneys revealed multiple expansile cysts containing proteinaceous substance, tubular atrophy, interstitial fibrosis and chronic inflammatory cell infiltration in keeping with PKD.

The cardiovascular pathology unit did not receive renal or other tissue sections or gross photographic documentation for independent review. The above renal findings are therefore based on the report and communication provided by the primary forensic pathologist.

## Discussion

Aortic dissection is a rare and fatal condition that usually occurs in people above 60 years old. Men are more prone to the condition. There are two key pathophysiology for the development of aortic dissection, the tear to the tunica intima leading to blood flowing into the false lumen or rupture of the small vessels that supply the aortic wall (vasa vasorum). Both conditions can exist concurrently [4].

Hypertension is one of the most common risk factors for aortic dissection in adults, whereas connective tissue and congenital cardiovascular anomalies are the common risk factors for persons below 35 years old. The common connective tissue diseases include Marfan’s, Ehlers–Danlos, and Loeys Dietz syndromes, whereas smoking, cocaine usage, bicuspid aortic valve, pregnancy, trauma, strenuous activity, previous heart surgery and atherosclerosis are other risk factors associated with aortic dissection [5].

Aortic dissection can be classified as Stanford Type A or B, or DeBakey Type I, II or III. Stanford Type A, DeBakey Type I and II involve the ascending aorta and arch and can involve the thoracoabdominal aorta. In contrast, Stanford Type B and DeBakey Type III involve the descending aorta or arch distal to the left subclavian artery [5]. The criteria for these classifications are based on the location and extent of the dissection or intimal tear.

While changes can be non-specific in older individuals, it is useful to perform histology in younger individuals where medial degeneration away from the dissection is supportive of a diagnosis of connective tissue disease. Haematoxylin and Eosin (H&E) or Elastic Van Gieson (EVG) staining can be done for microscopic examination. EVG staining is more beneficial because of its ability to demonstrate pathologies in the elastic fibers of the aortic wall.

ADPKD is a common hereditary disease. The occurrence of thoracic aorta dissecting aneurysm and abdominal aneurysm rupture predominates among males [6, 7]. Aortic aneurysm and dissection have been reported to occur more frequently in patients with ADPKD [8]. Besides that, multiple dissections may also involve the splenic and mesenteric arteries [9]. The occurrence of aortic dissection among young males is rare but has been reported previously, involving a 26-year-old [10].

Polycystin 1 (PKD 1) and Polycystin 2 (PKD 2) are two of the main gene mutations detected in ADPKD, which are present in the vascular endothelium and smooth muscles. The occurrence of the PKD 1 mutation in ADPKD patients is roughly 85–90%, whereas the PKD 2 mutation is between 10–15% [11].

Both PKD 1 and PKD 2 are responsible for membrane protein-coding, such as in calcium channel transport. The cellular proliferation and cyclic adenosine monophosphate (cAMP) increase when calcium channel abnormalities occur, leading to vascular wall damage. In addition, patients with ADPKD will also have a higher concentration of vasopressin, contributing to the rise of cAMP.

Besides that, the presence of cysts can contribute to renal parenchyma ischemia because the renal vessels can be constricted, henceforth activating the renin–angiotensin–aldosterone system. Activation of this system leads to hypertension, directly contributing to vascular wall damage.

The damage to the vascular wall can lead to an intracranial aneurysm, cervical artery dissection, coronary artery aneurysm, intracranial arterial dolichoectasia (dilatative arteriopathy) or an aortic aneurysm or dissection, which may represent associated vascular manifestations of ADPKD rather than direct causation, as elucidated in this case. The reported occurrence of aortic and abdominal aorta aneurysms is about 5–10% [11]. Besides that, PKD can also lead to liver cysts, characteristic of ADPKD, but it is frequently not present in younger individuals, such as in our case.

## History of aortic dissection and polycystic kidney disease

Aortic dissection was first reported in 1760 when King George II sustained a fatal cardiac tamponade due to a Type A aortic dissection. After roughly a century, in 1954, DeBakey, Cooley and Creech were the first to perform a successful surgical resection of a dissecting thoracic aortic aneurysm. The modern recognition and treatment of aortic dissection was led by 2 major developments. The first was the creation of the International Registry of Acute Aortic Dissection in 1996, and the issue of the *New England Journal of Medicine on* the 2nd of May 1999 [12].

Polycystic kidney disease was first defined in the eighteenth century when the Polish King Stephen Bathory died after complaining of chest pain and fatigue after a hunting trip. The examination of his kidneys during the mummification process revealed multiple cysts. The term polycystic kidney was first used in 1888 by Flix Lejars, and by the end of the nineteenth century, clinical signs, symptoms and genetic association were better understood [13].

## Conclusion

This case report intends to discuss the occurrence of aortic dissection in a previously healthy teenager who died unexpectedly with an incidental finding of PKD. Multiple factors could have led to this dissection, as aforementioned.

The cause of death was corroborated as aortic dissection. The question that had to be answered was the underlying cause of the aortic dissection. Trauma, accidental, suicidal and substance-related causes were excluded after the post-mortem examination. Hence, a natural causation is the most likely reason.

The post-mortem and histology examination revealed concentric left ventricular hypertrophy due to pressure overload, consistent with chronic hypertension. The primary causes of hypertension were not evident during the post-mortem and histology examination; hence, a secondary causation should be searched for. The presence of PKD, which is a recognized cause for secondary hypertension, can lead to vascular damage, progressive aortic medial degeneration and weakening predisposing to intimal tear and Stanford type A aortic dissection.

The finding of PKD was a significant piece of information. Furthermore, increased physical activity by the deceased during sexual intercourse can lead to an increased risk of aortic dissection if the aortic wall was previously weakened by PKD-associated damage.

Although other characteristic extra-renal features of PKD, such as visceral arterial aneurysm or hepatic cysts, were not seen, their absence should not prevent vascular complications associated with hypertensive damage.

The family was advised to undergo screening for both PKD and connective tissue disease, given the diagnosis. Although the pathological circumstances are rare, this case is a reminder that extremely uncommon manifestations can transpire in young individuals. Therefore, a meticulous approach should always be adopted before corroborating a cause of death.

## Data Availability

Data sharing does not apply to this article as no datasets were generated or analyzed during the current study.
